# A1BG and C3 are overexpressed in patients with cervical intraepithelial neoplasia III

**DOI:** 10.3892/ol.2014.2195

**Published:** 2014-05-28

**Authors:** NORMA ANGÉLICA GALICIA CANALES, VICENTE MADRID MARINA, JORGE SALMERÓN CASTRO, ALFREDO ANTÚNEZ JIMÉNEZ, GUILLERMO MENDOZA-HERNÁNDEZ, ELIZABETH LANGLEY McCARRON, MARGARITA BAHENA ROMAN, JULIETA IVONE CASTRO-ROMERO

**Affiliations:** 1 Research Center on Infection Diseases, National Institute of Public Health, Cuernavaca, Morelos 62100, Mexico; 2 Epidemiology and Health Services Research Unit, National Institute of Social Security, Cuernavaca, Morelos 62450, Mexico; 3 Laboratory of Peptides and Proteins, Department of Biochemistry, Faculty of Medicine, National Autonomous University of Mexico, Mexico City 04510, Mexico; 4 Biomedical Cancer Research Unit, Basic Research Subdirection, National Institute of Cancer, Mexico City 14080, Mexico

**Keywords:** cervical cancer, HPV, CIN III, complement C3, A1BG

## Abstract

The present study aimed to analyze sera proteins in females with cervical intraepithelial neoplasia, grade III (CIN III) and in healthy control females, in order to identify a potential biomarker which detects lesions that have a greater probability of cervical transformation. The present study investigated five sera samples from females who were Human Papilloma Virus (HPV) 16^+^ and who had been histopathologically diagnosed with CIN III, as well as five sera samples from healthy control females who were HPV-negative. Protein separation was performed using two-dimensional (2D) gel electrophoresis and the proteins were stained with Colloidal Coommassie Blue. Quantitative analysis was performed using ImageMaster 2D Platinum 6.0 software. Peptide sequence identification was performed using a nano-LC ESIMS/MS system. The proteins with the highest Mascot score were validated using western blot analysis in an additional 55 sera samples from the control and CIN III groups. The eight highest score spots that were found to be overexpressed in the CIN III sera group were identified as α-1-B glycoprotein (A1BG), complement component 3 (C3), a pro-apolipoprotein, two apolipoproteins and three haptoglobins. Only A1BG and C3 were validated using western blot analysis, and the bands were compared between the two groups using densitometry analysis. The relative density of the bands of A1BG and C3 was found to be greater in all of the serum samples from the females with CIN III, compared with those of the individuals in the control group. In summary, the present study identified two proteins whose expression was elevated in females with CIN III, suggesting that they could be used as biomarkers for CIN III. However, further investigations are required in order to assess the expression of A1BG and C3 in different pre-malignant lesions.

## Introduction

Cervical cancer is the third most common type of cancer in the female population worldwide. Globally, cervical cancer is considered to be the seventh most common type of cancer, with 530,232 cases reported in 2008. More than 85% of cases occur in developing countries (1). Latin America and the Caribbean have a very high incidence of cervical cancer. The World Health Organization reports 33,000 new cases per year (2,3). At present, in Mexico, cervical cancer is the second most common cause of cancer-associated mortality in females, and there is a population of 40.06 million 15-year old females who are at risk of developing cervical cancer. It is estimated that each year, 13,960 females are diagnosed with cervical cancer and that 4,476 succumb due to the disease (4). Cervical cancer develops due to persistent infection with the oncogenic Human Papilloma Virus (HPV), which progresses into an intraepithelial lesion, then into invasive cervical cancer.

The majority of patients are diagnosed at an advanced stage of cervical cancer, losing the most important window for treatment. This cancer is preceded by precursor lesions which have been classified into three progressive degrees, termed cervical intraepithelial neoplasia (CIN) I–III by Richart (5,6), while the Bethesda classification system divides the precursor lesions into low-grade squamous intraepithelial lesions (LSILs) and high-grade squamous intraepithelial lesions (HSILs) (7). HSILs have been proposed to be the true precursors to squamous cell cervical carcinoma. A number of studies have reported that persistent HSILs develop into carcinomas in 40–100% of cases, while LSILs show spontaneous regression (8,9).

The most common diagnostic method that is used to detect these lesions is based on the cytology and histopathology of the cervical tissues and cells. The Papanicolaou (PAP) smear, also known as the PAP test, is a low-cost method that is easily accessible, with 50% sensitivity and high susceptibility to intra- and inter-individual variability (10,11). This technique has many limitations, with false negative results often reported (20–30%) due to sample manipulation and contamination (12). The introduction of liquid-based cytology has contributed to a reduction in the efficiency problem associated with sample processing; however, validation in terms of sensitivity and specificity still presents deficiencies (10,11,13). Liquid-based cytology enables the detection of low- and high-grade dysplasia. Despite not being a definitive diagnostic method, liquid-based cytology does determine the presence of a lesion, as well as its topography, extension and severity. It also allows direct biopsies to be taken for further histopathological analysis (14).

In the last few years, there has been a trend toward identifying novel molecular biomarkers using proteomic tools that enable the identification of early lesions that have the greatest risk of malignant transformation. These types of tools allow the screening of proteins on a larger scale and from different biological samples, including serum, plasma, cells and tissues. They also allow the identification of molecules expressed at very low concentrations (fentomoles) with high sensitivity and specificity (15,16). Biofluids, including serum and plasma, are the main source of biomarkers due to their low cost, ease of collection, non-invasive collection and their easy processing (17,18).

A number of studies have investigated novel biomarkers in the serum (19–22), plasma (23–25) and tissue (26–31) from patients with early cervical lesions and/or cervical cancer, using proteomic tools. A large number of proteins have been identified that show differential expression between samples from healthy females and those from females with different types of intraepithelial lesions or cervical cancer. However, none of the proteins comply with the characteristics required for a specific marker, according to international requirements, which would be useful for the detection of lesions that have a greater probability of being transformed into cancer. At present, the markers under validation are Ki67, pINK4A, MIB-1 and ProExC (32–34). The present study aimed to investigate a marker in the serum of females infected with HPV and the histopathological diagnosis of advanced CIN (CIN III).

## Materials and methods

### Patients and biological samples

The present study is a descriptive and transversal pilot study on serum samples from a population of Mexican females. A total of 10 samples (five controls and five cases) of blood were obtained from females aged between 28 and 65 years who were recruited from the Hospital General Regional del Estado de Morelos (IMSS; Cuernavaca, Mexico) who fulfilled the inclusion criteria and accepted to donate a blood sample under informed consent. The present study was reviewed and approved by the Ethics Committee of the IMSS and the IRB from the Instituto Nacional de Salud Pública (Cuernavaca, Mexico). The inclusion criteria for the cases were: (i) a positive Hybrid Capture test; (ii) an abnormal PAP test; and (iii) CIN III lesion diagnosis confirmed via colposcopy and histopathology, using the Bethesda criteria. Colposcopic and histopathologic analyses were performed by two specialists from the Colposcopy Unit at the IMSS and two pathologists form the Pathology Unit at the IMSS. In addition, positivity for HPV 16 was confirmed using polymerase chain reaction (PCR) analysis (Table I). Only the serum samples from the females who were diagnosed with CIN III were included in the present study. The inclusion criteria for the control group were: (i) a negative hybrid capture test; (ii) a normal PAP test; and (iii) no apparent injury through gynecological physical examination. For the control and case groups, the inclusion criteria also included not having had a HPV vaccine, not having taken oral contraceptives in the previous six months and not having received chemotherapy.

### Blood sample processing

Blood samples (3 ml) were obtained using venipuncture in Vacutainer^®^ SSTTM tubes (Becton-Dickinson, Mexico City, Mexico) with separator gel and were left at room temperature for 1 h. Samples were then centrifuged at 1,300 × g for 15 min. The serum was stored at −80°C in 100 μl aliquots until processed. Serum aliquots (10 μl) in triplicate from each patient were passed through a high-affinity column to remove albumin and immunoglobulin G (IgG) using a commercial kit (ProteoSeek™ 162 Albumin/IgG removal kit; Pierce Biotechnology Inc., Rockford, IL, USA). Other interfering substances, including detergents, salts, lipids, nucleic acids and phenolic acids, were removed from the samples using the Two-dimensional (2D) Clean Up kit (GE Healthcare; Little Chalfont, UK). Serum protein quantification was performed using the 2D Quant kit (GE Healthcare).

### 2D gel electrophoresis (2D-GE)

A total of 200 μg protein (in triplicate) was mixed with rehydration buffer [8 M urea, 2% CHAPS (Roche Diagnostics GmbH, Mannheim, Germany), 0.5% IPG2 buffer (pH 3–10; GE Healthcare), 0.002% bromophenol blue and 0.56 M dithiothreitol (DTT; Sigma-Aldrich, Munich, Germany)] in a final volume of 250 μl. To each tube, 1 μl IPG2 buffer and 0.00056 g DTT was added. Isoelectric focusing was performed using an Ettan™ IPGphor™ unit using 13-cm strips (GE Healthcare). The rehydration time was 14 h at a constant current of 50 mA per strip. The voltages used for isoelectric focusing were 500, 1,000 and 8,000 V/h. The strips were then equilibrated in buffer [50 mM Tris-HCl (pH 8.8; Sigma-Aldrich), 6 M urea, 30% Glycerol, 2% SDS (Sigma-Aldrich), 0.002% bromophenol blue (Sigma-Aldrich) and Milli-Q™ water (Millipore Milli-Q lab water system; Millipore, Billerica, MA, USA)] supplemented with DTT (50 mg/5 ml) for 15 min. The strips were then equilibrated with iodoacetamide (125 mg/5 ml; GE Healthcare) for the same duration of time. Protein separation was performed using SDS-PAGE in two dimensions on gradient gels of 7.5–20% at a constant voltage of 150 V for 5.5 h. Gels were stained with Colloidal Coomassie Blue G-250 (Bio-Rad, Hercules, CA, USA), as described previously (34). Subsequent to staining, the gels were scanned using LabScan™ 5.0 (GE Healthcare). Digital images were used for detection and analysis using ImageMaster 2D Platinum 6.0 software (GE Healthcare). The selected spots were identified using electrospray ionization time-of-flight mass spectrometry (ESI-TOF-MS). In brief, protein spots were excised from the Coomassie-stained 2D gels and destained using 50% (v/v) methanol and 5% (v/v) acetic acid (Mallinckrodt, Baker Inc., Paris, KY, USA) for 12 h. The destained gels were then washed with deionized water, soaked for 10 min in 100 mM ammonium bicarbonate (Sigma-Aldrich), cut into small pieces, completely dehydrated using 100% acetonitrile (Mallinckrodt, Baker Inc.) and vacuum-dried. In-gel digestion was performed through adding 30 μl modified porcine trypsin solution (20 ng/ml; Promega Corporation, Madison, WI, USA) to 50 mM ammonium bicarbonate followed by overnight incubation at room temperature. Peptides were extracted using 50% (v/v) acetonitrile and 5% (v/v) formic acid (Mallinckrodt, Baker Inc.) twice for 30 min with sonication. The extract volumes were reduced using evaporation in a vacuum centrifuge and were adjusted to 20 μl using 1% (v/v) formic acid.

Liquid chromatography tandem mass spectrometry (*LC*/*MS*/*MS).* Mass spectrometric analysis of the tryptic peptides was performed using an integrated nano-LC ESI MS/MS system (Synapt G2 High Definition mass spectrometer; Waters Corporation, Milford, MA, USA) equipped with a NanoLockSpray™ ion source. The instrument was coupled online to a NanoAcquity Ultra Performance liquid chromatograph (UPLC; Waters Corporation). The binary solvent system used was 2% acetonitrile in Milli-Q water with 0.1% formic acid (mobile phase A) and 98% acetonitrile in Milli-Q water with 0.1% formic acid (mobile phase B). Samples were concentrated and desalted through injection into a Symmetry C18 UPLC trapping column (5 mm, 180×20 mm; Waters Corporation) and washed with 100% mobile phase A at a flow rate of 15 μl/min for 3 min. Next, the trap column was switched in-line (coupled) with the analytical BEH C18 UPLC column (1.7 μm, 75 μmx100 mm; Waters Corporation) for peptide seperation, using a linear gradient to 40% B over a 30 min period, at a flow rate of 0.3 μl/min. The column was then washed for 10 min with 98% mobile phase B.

The mass spectrometer was calibrated using an NaCl solution and operated in ESI positive V-mode at a resolution of 10,000 full width at half height. Spectra were acquired in the automated mode using data-dependent acquisition (DDA). Fibrin peptide B solution (100 fmol/μl) was infused through the reference sprayer of the NanoLockSpray source at a flow rate of 500 nl/min and was sampled at 30 sec intervals during the acquisition. MS survey scans of 1 sec over the m/z range 300–1,600 were used for the peptide detection followed by two MS/MS scans of 2 sec each (m/z, 50–2,000) of detected precursors. Collision energies were automatically adjusted based on the ion charge state and the mass. The five most intensive precursor ions were interrogated per MS/MS switching event. Dynamic exclusion for 60 sec was used in order to minimize multiple MS/MS events for the same precursor.

### Data processing and protein identification

DDA raw data files were processed and converted to pkl files using ProteinLynx Global Server software, version 2.4 (Waters Corporation). Pkl files were subsequently database-searched using the Mascot search algorithm (version 1.6b9; Matrix Science, London, UK). The specific genome was not available; thus, searches were performed using the human subset of the National Center for Biotechnology Information non-redundant database (NCBInr; http://www.ncbi.nih.gov). Trypsin was used as a specific protease and one missed cleavage was allowed with mass tolerances of 50 ppm and 0.05 Da for the precursor and fragment ion, respectively. Variable modifications included methionine oxidation and glutamine-asparagine deamination. Peptide matches with Mascot scores exceeding the 95% level of confidence were accepted as correct matches. The threshold score was 48 for P<0.05.

### Western blot analysis for α-1-B glycoprotein (A1BG) and complement component 3 (C3)

The serum samples used for western blot analysis were obtained from a group of 55 females participating in the HPV Detection Service at the IMSS. This group of females underwent a Pap smear and a hybrid capture test. If the Pap smear showed any alterations, the participants also underwent colposcopic and histopathologic analysis. For the western blot analysis, the control group (n=30) included females with a normal Pap smear and a negative hybrid capture test, while the case group (n=25) included females with an abnormal Pap smear, positive hybrid capture test and a diagnosis of CIN III using colposcopy and histopathology. In addition, in the case group, the HPV type was identified using PCR (HPV 16, 56%; HPV 58, 8%; HPV 33, 8%; HPV 18, 4%; and unidentified, 24%). A total of 20 μg total protein from the serum was separated using unidimentional 10% SDS-PAGE and transferred to polyvinylidene fluoride membranes (GE Healthcare). The membranes were blocked for 1 h in 0.1% Tween 20 and 5% non-fat dry milk in Tris-buffered saline (TBS) at room temperature. Primary antibodies against mouse monoclonal Ig-G anti-human A1BG (clone 51A6) and mouse monoclonal Ig-G anti-human complement C3 (clone 2898) (Santa Cruz Biotechnology, Inc., Santa Cruz, CA, USA) were diluted 1:2,000 and 1:1,000, respectively in 0.1% Tween 20 and 5% non-fat dry milk in TBS. The membranes were then incubated with the primary antibodies overnight at 4°C. Membranes were subsequently washed with 0.1% Tween 20 in TBS and incubated with polyclonal goat anti-mouse IgG-horseradish peroxidase secondary antibodies (Santa Cruz Biotechnology, Inc., Santa Cruz, CA, USA) for 1 h at a dilution of 1:2,000. Peroxidase activity was visualized using colorimetry with 3,3′,5,5′-tetramethylbenzidine (Invitrogen Life Technologies, Carlsbad, CA, USA). Subsequent to immunodetection, membranes were washed twice with TBS and stained with 0.1% Coommassie R-250 (GE Healthcare), which was used as a loading control, according to the method described by Welinder and Ekblad (36). Densitometric quantification of the western blots was determined using Image J software (National Institutes of Health, Bethesda, MA, USA).

Even though the haptoglobins and apolipoproteins identified by spectrometry showed higher scores than the complement C3 and A1BG, only the latter was analyzed. This was due to the evidence that, in 2008, Barba de la Rosa *et al* (20) had shown increases in serum haptoglobins in females with different degrees of cervical cancer lesions. There is also little information with regard to the possible role of C3 and A1BG in this type of cancer, particularly in precursor lesions.

### Detection and typification of viral DNA

Cervical cells were obtained using endocervical curettage and collected in transport medium (HC2 DNA Collection Device; Digene, Gaithersburg, MD, USA). Samples were transported and stored at −20°C until use. The presence of infection with oncogenic types was determined through hybrid capture using the Hybrid Capture II kit (Digene), according to the manufacturer’s instructions, which detects the following HPV oncogenic types: 16, 18, 31, 33, 35, 39, 45, 51, 52, 56, 58, 59 and 68 (37). Positive samples were used for viral typification using PCR analysis. In brief, extraction and purification of DNA was performed using the Genomic DNA Purification kit (Fermentas, Burlington, ON, Canada) according to the manufacturer’s instructions. DNA integrity was determined through amplifying a 450-bp fragment of the constitutive GAPDH gene using the following oligonucleotide sequences: Forward, 5′-ACC ACA GTC CAT GCC ATC AC-3′ and reverse, 5′-TCC ACC ACC CTG TTG CTG TA-3′. Verification of the presence of the HPV L1 gene was performed using PCR amplification of a 450-bp fragment using the MY11 (5′-GCM CAG GGW CAT AAY AAT GG-3′) and MY09 (5′-CGT CCM ARR GGA WAC TGA TC-3′) oligonucleotide sequences. DNA samples which amplified the HPV L1 fragment were analyzed using restriction fragment length polymorphism to typify the HPV (38). The samples were digested with four restriction endonucleases (*Rsa*1*, Acc* I*, Dde* I and *Xba*I), and incubated at 30°C for 2 h. The band profile was visualized on 6% polyacrylamide gels. SiHa and CasKi cells were used for the DNA positive control, while water was used as a negative control. Viral type confirmation was determined through sequencing using specific oligonucleotides for each viral type.

## Results

### Sample characteristics

Serum protein profiles were analyzed in samples from females in the control group (n=5) and those diagnosed with CIN III through histopathological analysis (n=5). The individuals in each group fulfilled their respective inclusion criteria. The range of ages, diagnosed oncogenic HPV infection, viral type and histological and cytological diagnoses are shown in Table I, which demonstrates the homogeneity of the analyzed samples. All of the females in the control group were found to have a normal PAP result and were negative for oncogenic viruses, while those in the case group had an abnormal PAP result, were positive for oncogenic virus HPV 16 and had a confirmed diagnosis of CIN III. The average age of the individuals in in the control and case groups was 42.6 and 45.4 years, respectively, with the two groups having a similar median age (41 and 42 years, respectively).

### 2D-GE

**Figure 1:**
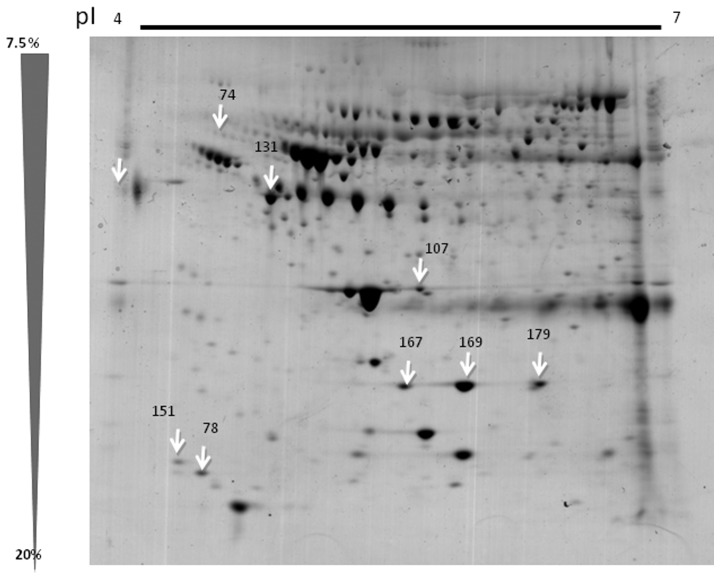
Two dimensional gel electrophoresis map of a serum sample from a patient with cervical intraepithelial neoplasia, grade III stained using Colloidal Coomassie Blue G-250. The white arrows indicate spots that were differentially expressed between the control and case groups. The numbers indicate the spot number assigned by Image Master Software (GE Healthcare, Little Chalfont, UK). pI, isoelectric point.

The serum from the individuals in the case and control groups was analyzed in triplicate using 2D SDS-PAGE and the differential expression was assessed using ImageMaster 2D Platinum 6.0 software (GE Healthcare). The program detected 337 spots that belonged to the healthy group and 516 spots that belonged to the cases diagnosed with CIN III. Matching between the groups resulted in 189 matches common to the two groups. Eight spots were selected for further identification (Fig. 1). The parameters used for the selection of these spots were that the spots were present in 8–11 gels, and that they were only present in the case group and not in the control group, indicating a constant presence of the spot. These spots were excised from the gels, digested and identified using ESI-TOF-MS analysis.

### LC-ESI-MS-MS analysis

The eight spots selected from the 2D gels were identified using ESI-TOF-MS analysis. The peak of the mass of the peptide was compared to that in the NCBInr database. Table II shows the proteins that were sequenced from the eight spots, which correspond to the A1B319 glycoprotein, C3, one pro-apolipoprotein, two apolipoproteins and three haptoglobins.

### Western blot analysis

**Figure 2:**
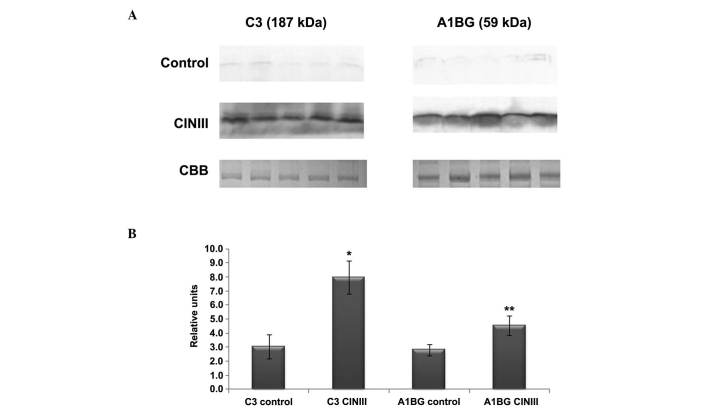
(A) Western blots detecting the protein expression of C3 and A1BG in 20 μg total protein in serum obtained from females in the control group (n=30) and those with CIN III (n=25). Specific antibodies were used to detect A1BG and C3 at a dilution of 1:2,000 and 1:1,000, respectively. (B) Densitometry analysis using Image J v 1.45. CBB staining was used as a loading control. Data are presented as the mean ± standard deviation. P<0.05 was considered to indicate a statistically significant difference. A1BG, α-1-B glycoprotein; C3, complement component 3; CBB, Coommassie Brilliant Blue; CINIII, cervical intraepithelial neoplasia, grade III.

Fig. 2A shows a representative western blot of the expression of A1BG and C3 in the serum of the females diagnosed with CIN III (n=25) and those in the healthy control group (n=30). Fig. 2B shows densitometry analysis of the corresponding bands from the two groups. The relative density of the A1BG and C3 protein bands was observed to be greater in the serum samples of the females with CIN III, compared with those in the control group. For C3, statistical analysis revealed that the expression values for the individuals in the control and CIN III groups were 3.1±0.85 and 8.0±1.17 (t=5.7; P≤0.05), respectively. For A1BG, the expression values were 2.8±0.40 and 4.5±0.68 in the control and CIN III groups (t=4.3; P≤0.05), respectively.

## Discussion

Proteins are encoded by the genes that constitute the human genome, and they are directly responsible for regulating cellular function through the activation or inactivation of different signaling pathways associated with cell proliferation, death and metabolism. It is well established that the number of proteins expressed in a cell at a particular time does not correspond with the total number of activated genes. Thus, it is necessary to detect the profile of the proteins expressed at specific times and under particular conditions in healthy subjects and those with particular pathologies. Furthermore, the protein profiles of the serum, plasma, tissue, urine and other human fluids may be modified as a result of acute or chronic diseases, either due to proteins produced by infectious agents, including viruses, bacteria and fungi, or due to alterations at the genetic level. The identification if these protein profiles has enabled the identification of biomarkers for certain pathologies, which may be used for disease prevention, diagnosis and follow-up (39). Certain biomarkers have been approved by the Food and Drug Administration for certain types of cancer (40).

Cervical cancer is a pathology whose causal agent is HPV, which modifies the intracellular biology of the host during infection and through its integration into the genome. In addition, HPV induces an immune response on local and systemic levels. A number of these changes are reflected in the expression profiles of certain proteins in the tissue, plasma, serum and cervical mucus of the infected patients. Our group has been interested in investigating these profiles in the serum, plasma and cervical mucus of females with different pre-malignant lesions and cervical cancer. The present study utilized proteomic tools to investigate serum proteins in females diagnosed with CIN III compared with healthy females.

The present study identified eight proteins with an increased expression in the serum of patients with CIN III lesions compared with healthy females. These eight proteins included A1BG, C3, a pro-apolipoprotein, two apolipoproteins and three haptoglobins. The serum expression of A1BG and C3 was confirmed using western blot analysis in 25 females with CIN III and 30 control individuals. The remaining six proteins were not analyzed in this manner, as previous studies have demonstrated their overexpression in the serum (20) and plasma (22) of females with different grades of intraepithelial lesions and cervical cancer. In the present study, western blot analysis revealed that A1BG and C3 were overexpressed in all of the females in the CIN III group compared with those in the control group, suggesting that these two proteins may participate in the regulation of a pre-malignant lesion-induced immune response or may be involved in carcinogenesis. However, further studies using other types of methodologies are required to investigate this hypothesis. Similar findings were reported by Jeong *et al* (24) who analyzed plasma samples from six healthy females compared with six females with squamous cell carcinoma (SCC) and identified that A1BG and C3 were overexpressed using 2D-GE analysis.

This increased expression of C3 was also confirmed and validated in this study in a group of females diagnosed with cancer *in situ*. However, the present study identified that C3 overexpression was present in earlier disease stages. Thus, it is important to elucidate the point at which C3 expression is initiated in order to determine whether this increase is associated with the presence of HPV during the infection stage and whether it may be useful as a biological marker.

C3 is a key protein in the complement cascade and its expression is essential for the activation of all three complement pathways (41). It is involved in the immune system and has a key role in destroying invasive microorganisms and cleaning up dead and apoptotic cells. The complement system is one of the most highly conserved cellular systems (42). Complement proteins act as zymogens, which are transformed into enzymes and activate complement proteins or different receptors. Elevated concentrations of complement proteins have been found in the serum of patients with ovarian cancer (43), hepatitis C-associated hepatocellular cancer (44), pancreatic cancer (45), small cell renal carcinoma (46) and SCC (24). In 1980, through simple immunodiffusion assays, Pulay *et al* (47) demonstrated that the average level of C3 increases with the progression of cervical cancer lesions up to stage III, but diminishes by stage IV (48). In contrast to these previous findings, fragments of C3 and C4 A/B have been reported to be reduced in the plasma of patients with SCC of the penis, and this reduction was found to become more evident as the disease progressed, suggesting that these fragments may be good candidates for prognostic tools (48). The cause of this reduction is unknown; however, it may be the result of infection with HPV or the Epstein Barr virus, which are prevalent in this type of cancer in males (49), and whose proteins may be attacking the immune system (50,51). However, this hypothesis has yet to be elucidated.

These contradictory findings suggest that complement proteins may be differentially regulated, depending on the type and origin of the cancer, thus further research is required. A previous study suggested that not only are there certain proteins with a high variability under normal conditions, including haptoglobin (0–40 mg/ml), lysozyme (0.01–0.1 mg/ml) and C-reactive protein (0,01–0,3 mg/ml), but also those that have a low variability, including albumin, which has a coefficient of variation (CV) of 9%, as well as transferrin 410 (CV, 14%), C3, (CV, 17%), α-1 acid glycoprotein (CV, 21%), α 2-macroglobulin (CV, 20%), transthyretin fragment (CV, 28.3%) and β-chain α 2-HS-glycoprotein (CV, 29,7%) (52), which may be important for determining the state of health of an individual. It is important to determine whether the changes in the expression of C3 and A1BG identified in the present study, are capable of providing insight into the changes that occur throughout the progression of cervical lesions, prior to their transformation into cervical cancer.

A1BG is a protein found in the serum and plasma whose function has yet to be elucidated. A1BG shows homology to the immunoglobulin family, through its duplication and its nucleic acid sequence (53). A1BG is present in normal adult plasma at a concentration of 22 mg/dl (54). However, the biological function of A1BG has yet to be elucidated and it has been found to be elevated in certain types of cancer (19). A1BG has been reported to be elevated in the serum of patients with endometrial cancer and cervical cancer (19), as well as patients with cervical squamous cell carcinoma (24,54). These findings suggest that A1BG may be involved in cervical carcinogenesis; thus. elucidating its function is important.

Haptoglobins are glycoproteins which are capable of binding hemoglobin and are secreted by hepatic cells in response to different stimuli and function as iron transporters and recyclers. Haptoglobin concentration depends on the level of hemoglobin and the greater the hemoglobin concentration, the lower the concentration of haptoglobins (55). It has been reported that iron favors the growth of cancer cells and that it preferentially accumulates in cancer cells compared with normal cells (56).

Thus, it has been suggested that haptoglobins may be potential markers for patients with ovarian, lung (57) and colon cancer (58). However, the role of haptoglobins in cervical cancer has yet to be elucidated, particularly as the difference in the expression of these proteins in females with different lesion grades (low grade, high grade and cancer) is very discreet as the lesions progress (20), suggesting that haptoglobins may not be good candidate biomarkers for this pathology. The present study found that three isoforms of haptoglobins were expressed in the individuals in the healthy control group, as well as those with CIN III, and that there was no significant difference in their expression between the two groups. Apolipoprotein (APO) C-III is a transporter molecule for high density lipoproteins which regulates different cellular proteins involved in oxidation, apoptosis, cellular recognition and transport (59). Apolipoproteins have been associated with different types of cancer, including hepatocellular cancer (60) and breast cancer (55). For example, ApoC-I has been found to be increased in colon, prostate and liver cancer, while ApoC-III has been reported to be associated with pancreatic, breast and colon cancer (61). However, the expression of apolipoproteins in patients with cervical cancer has yet to be elucidated.

In conclusion, serum may be alternative source for investigating differential protein profiles between healthy females and those with HSILs, for example CIN III, secondary to HPV infection. A number of studies, reviewed by Rutkowski *et al* (51) and Pio *et al* (62), suggest that modifications in the complement system may contribute to tumor development due to the influence of these proteins on processes involving proliferation, angiogenesis, invasion, migration and survival. Thus, it is possible that in the case of cervical cancer, complement system proteins, including C3, may act as coadjuvants in the development of lesions into the disease. However, further investigations are required to elucidate the mechanisms involved and to determine whether other proteins from the same family are associated with this type of cancer.

## Figures and Tables

**Table I:** Characteristics of the individuals in the case and control groups who were included for 2D-gel analysis.

Sample	Number	Age (years)	PAP test	Hybrid capture test	Colposcopy	Histopathology	HPV type
Control group							
1	C1-IMSS	38	Normal cytology	(−)	Nd^a^	Nd^a^	Nd
2	C2-IMSS	41	Normal cytology	(−)	Nd^a^	Nd^a^	Nd
3	C3-IMSS	47	Normal cytology	(−)	Nd^a^	Nd^a^	Nd
4	C4-IMSS	47	Normal cytology	(−)	Nd^a^	Nd^a^	Nd
5	C7-IMSS	40	Normal cytology	(−)	Nd^a^	Nd^a^	Nd
Case group							
1	T1-IMSS	65	Abnormal cytology	(+)	HSIL	CIN III	16
2	T3-IMSS	60	Abnormal cytology	(+)	HSIL	CIN III	16
3	T7-IMSS	42	Abnormal cytology	(+)	HSIL	CIN III	16
4	T8-IMSS	32	Abnormal cytology	(+)	HSIL	CIN III	16
5	T10-IMSS	28	Abnormal cytology	(+)	HSIL	CIN III	16

Nd^a^, not done due to ethical considerations and lack of medical indication. HPV, Human Papilloma Virus; IMSS, Hospital General Regional del Estado de Morelos Del Estado de Morelos; PAP, Papanicolaou; Nd, not done due to negative hybrid capture test; HSIL, high-grade squamous intraepithelial lesion; CIN III, cervical intraepithelial neoplasia, grade III.

**Table II:** Proteins analyzed using ESI-TOF-MS and identified using the Mascot search algorithm.

Spot number	Access number^a^	Description	Function	Score	Peptide matched^b^	Coverage (%)	MW (kDa)	pI
74	gi69990	**α-1-B-glycoprotein**	Unknown	96	6 (2)	5 (2)	51,908	6.23
78	gi78101271	**Complement component 3**	Immune system	128	18 (3)	13 (5)	39,463	6.23
107	gi178775	Pro apolipoprotein	Trygliceride components and high lipoproteins	474	36 (12)	13 (9)	28,944	6.23
131	gi52120	Apolipoprotein	Trygliceride components and high lipoproteins	474	36 (12)	13 (9)	28,944	5.23
151	gi521205	Apolipoprotein C III	Trygliceride components and high lipoproteins	57	5 (1)	3 (1)	10,815	5.65
167	gi22397	Haptoglobin H2	Antioxidant, angiogenesis inductor, antiinflamatory effect	142	13 (3)	8 (4)	41,717	5.45
169	gi223976	Haptoglobin H2	Antioxidant, angiogenesis inductor, antiinflamatory effect	204	21 (4)	100	41,717	5.45
179	gi296653	Haptoglobin H2	Antioxidant, angiogenesis inductor, antiinflamatory effect	142	13 (3)	100	41,499	5.00

Bold text denotes the possible candidate proteins specific for cervical intraepithelial neoplasia, grade III.

a From SwissProt database;

b data are presented as the experimental value (predicted value).

ESI, electrospray ionization; TOF, time-of-flight; MS, mass spectrometry; MW, molecular weight; pI, isoelectric point.
